# DNA of Piroplasms of Ruminants and Dogs in Ixodid Bat Ticks

**DOI:** 10.1371/journal.pone.0167735

**Published:** 2016-12-08

**Authors:** Sándor Hornok, Krisztina Szőke, Dávid Kováts, Péter Estók, Tamás Görföl, Sándor A. Boldogh, Nóra Takács, Jenő Kontschán, Gábor Földvári, Levente Barti, Alexandra Corduneanu, Attila D. Sándor

**Affiliations:** 1 Department of Parasitology and Zoology, University of Veterinary Medicine, Budapest, Hungary; 2 Department of Evolutionary Zoology and Human Biology, Debrecen University, Debrecen, Hungary; 3 Department of Zoology, Eszterházy Károly University, Eger, Hungary; 4 Department of Zoology, Hungarian Natural History Museum, Budapest, Hungary; 5 Department of Nature Conservation, Aggtelek National Park Directorate, Jósvafő, Hungary; 6 Plant Protection Institute, Centre for Agricultural Research, Hungarian Academy of Sciences, Budapest, Hungary; 7 Romanian Bat Protection Association, Satu Mare, Romania; 8 Department of Parasitology and Parasitic Diseases, University of Agricultural Sciences and Veterinary Medicine, Cluj-Napoca, Romania; Onderstepoort Veterinary Institute, SOUTH AFRICA

## Abstract

In this study 308 ticks (*Ixodes ariadnae*: 26 larvae, 14 nymphs, five females; *I*. *vespertilionis*: 89 larvae, 27 nymphs, eight females; *I*. *simplex*: 80 larvae, 50 nymphs, nine females) have been collected from 200 individuals of 17 bat species in two countries, Hungary and Romania. After DNA extraction these ticks were molecularly analysed for the presence of piroplasm DNA. In Hungary *I*. *ariadnae* was most frequently identified from bat species in the family Vespertilionidae, whereas *I*. *vespertilionis* was associated with Rhinolophidae. *Ixodes ariadnae* was not found in Romania. Four, four and one new bat host species of *I*. *ariadnae*, *I*. *vespertilionis* and *I*. *simplex* were identified, respectively. DNA sequences of piroplasms were detected in 20 bat ticks (15 larvae, four nymphs and one female). *I*. *simplex* carried piroplasm DNA sequences significantly more frequently than *I*. *vespertilionis*. In *I*. *ariadnae* only *Babesia vesperuginis* DNA was detected, whereas in *I*. *vespertilionis* sequences of both *B*. *vesperuginis* and *B*. *crassa*. From *I*. *simplex* the DNA of *B*. *canis*, *Theileria capreoli*, *T*. *orientalis* and *Theileria* sp. OT3 were amplified, as well as a shorter sequence of the zoonotic *B*. *venatorum*. Bat ticks are not known to infest dogs or ruminants, i.e. typical hosts and reservoirs of piroplasms molecularly identified in *I*. *vespertilionis* and *I*. *simplex*. Therefore, DNA sequences of piroplasms detected in these bat ticks most likely originated from the blood of their respective bat hosts. This may indicate either that bats are susceptible to a broader range of piroplasms than previously thought, or at least the DNA of piroplasms may pass through the gut barrier of bats during digestion of relevant arthropod vectors. In light of these findings, the role of bats in the epidemiology of piroplasmoses deserves further investigation.

## Introduction

Bats (Chiroptera) are the second largest order of mammals, with more than 1200 species. Owing to their flying habit, feeding preference and high level of adaptability, bats are widely distributed and present on all continents except the Antarctica [1]. In this scenario, invasion of men into bat habitats and adaptation of bats to urban areas increased the chances for contact between humans, domestic animals and bats [2], thus promoting the chances of pathogen transmission.

Accordingly, during the past decades, the epidemiological significance of bats has become increasingly recognized. Bats can be reservoirs or carriers of numerous species of viruses, bacteria and parasites, among them many with zoonotic potential to infect humans [3]. This is especially important in the context of synanthropic life of several bat species, which tend to roost or breed in human settlements, even in man-made buildings such as steeples, attics, cellars and barns [4]. In comparison with bat species that prefer forested habitats, urbanized populations of chiropterans may form larger, more stable and aggregated colonies [1,4]. Thus, urban bats may reach the highest individual number in the local mammalian fauna, further increasing their epidemiological significance. In addition, bat species that are indigenous in Europe feed predominantly on arthropods, and this makes it possible for them to get into contact with vector-borne pathogens not only from their ectoparasites, but also from their food [5].

Blood-sucking ectoparasites of bats may be potential vectors of a broad range of pathogens [2]. Depending on their taxonomic group, these ectoparasites can be highly (e.g. bat flies) or less specialized to bats (e.g. soft ticks, bugs), and exceptionally can settle on other mammals, even on humans [6]. Interestingly, while ixodid ticks of bats are not known to feed on other mammals, except *Ixodes vespertilionis* on humans [7], ixodid ticks that frequently infest domestic animals (e.g. *Ixodes ricinus*, *Dermacentor reticulatus*, *Haemaphysalis* spp.) have also been collected from bats [8, 9, 10].

Three species of ixodid bat ticks (Acari: Ixodidae) occur in Europe, i.e. *I*. *vespertilionis*, *I*. *ariadnae* and *I*. *simplex* [11]. These are specialized for bats, i.e. all three developmental stages that need a blood meal (larvae and nymphs for moulting, females for oviposition) will typically suck blood on bats, as exemplified by *I*. *ariadnae* [11]. While these three tick species and their genotypes appear to be widely distributed across the Palaearctic [12], only few data are available on their vector potential. Concerning molecular investigations of vector-borne pathogens in ixodid bat ticks, bartonellae were reported from *I*. *vespertilionis* [13], but to the best of our knowledge none from *I*. *simplex* or *I*. *ariadnae*. Therefore the present study was undertaken to ameliorate this lack of data on pathogens and/or pathogen DNA carried by ixodid bat ticks. Recently, DNA of *Babesia canis* has been detected in bat faeces [5], therefore piroplasms were chosen as the target group of analyses. It was also within the scope of this study to examine the geographical range and host spectrum of these tick species in Hungary and Romania, by including 17 species of bats (from three families and six genera) from these two countries.

## Materials and Methods

### Tick collection and identification

**Fig 1:**
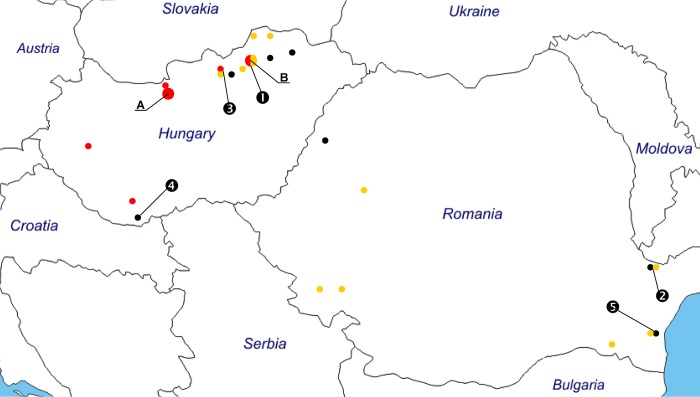
Sampling sites of the present study. Color of collection sites for *Ixodes ariadnae* are marked with red dots, for *I*. *vespertilionis* with yellow dots, and for *I*. *simplex* with black dots. Letters: A—Ariadne Cave System and caves in the Pilis Mountains (bats sampled at three caves), B—Bükk Highlands Cave system (bats sampled at nine caves). Numbers in black circles indicate places, where piroplasm-carrier bat ticks were collected (Table 3). Small and unseparated dots with different colour indicate the same place with two tick species. Two places close to each other in northeast Hungary (Baradla and Béke Caves) are marked with one dot. Coordinates for the individual places are shown in S1 Table.

Ixodid ticks were collected from bats, caught for monitoring purposes, on 24 locations in Hungary in 2008–2015, and on seven locations in Romania in 2015 (Fig 1; data in S1 Table). These bats were caught (as part of a monitoring program) at the entrance of caves between sunset and dawn, using standard Ecotone mist-nets (Gdynia, Poland) with 12 m length, 2.5 m height and 14 × 14 mm mesh. Bats were handled without anesthesia, but released immediately after tick removal to alleviate suffering. Data (species, sex) of bats, from which the ticks were removed, were recorded. The ticks were immediately put into and stored in 96% ethanol. Morphological identification was done with a stereo microscope (SMZ-2T, Nikon Instruments, Japan) using standard morphological keys (subadults: [14]; females: [15]).

### DNA extraction and molecular analyses

DNA was extracted (except for one *I*. *simplex* larva from *Barbastella barbastellus*) with the QIAamp DNA Mini Kit (QIAGEN, Hilden, Germany) according to the manufacturer's instructions and including extraction controls. Ticks were dried, then washed three times (in detergent containing water, in tap water and in distilled water) and minced at the bottom of 1.5 ml Eppendorf tubes in 100 μl PBS with pointed scissors. Between each sample the scissors were washed and burned for decontamination. Samples were then incubated overnight at 56°C in tissue lysis buffer containing proteinase-K.

DNA samples were molecularly screened with a conventional PCR that amplifies an approx. 500 bp long part of the 18S rDNA gene of piroplasms, modified from Casati et al. [16]. The primers BJ1 (forward: 5'-GTC TTG TAA TTG GAA TGA TGG-3') and BN2 (reverse: 5'-TAG TTT ATG GTT AGG ACT ACG-3') were used in a reaction volume of 25 μl, which included 5 μl of extracted DNA, and 20 μl of reaction mixture containing 0.5 unit HotStarTaq Plus DNA polymerase (5U/ μl), 200 μM PCR nucleotid mix, 1 μM of each primer and 2.5 μl of 10× Coral Load PCR buffer (15 mM MgCl_2_ included). For amplification an initial denaturation step at 95°C for 10 min was followed by 40 cycles of denaturation at 95°C for 30 s, annealing at 54°C for 30 s and extension at 72°C for 40 s. Final extension was performed at 72°C for 5 min.

All PCRs were run with appropriate positive and negative controls. During all tests positive controls showed positivity, whereas negative (non-template) controls and extraction controls remained negative (the latter indicating absence of sample contamination). PCR products were electrophoresed in a 1.5% agarose gel, stained with ethidium-bromide and visualized under ultra-violet light. Purification and sequencing (twice) were done from all PCR positive samples by Biomi Inc. (Gödöllő, Hungary). Representative sequences were submitted to GenBank (accession numbers KU958544-53). Phylogenetic analyses were conducted according to the Tamura-Nei model [17] and Maximum Composite Likelihood method by using MEGA version 5.2 [18].

### Statistical analyses

Association of tick species with bat families was assessed by Fisher's exact test. Intensities of tick infestation (i.e. number of ticks on a bat individual) were compared between bat species by using Mann-Whitney U-test and Kruskal Wallis H-test in R program. The following bat species (in Table 1, Table 2: harboring different stages of the same tick species) were analysed: *Myotis bechsteinii* (n = 7), *My*. *daubentonii* (n = 28), *My*. *emarginatus* (n = 9), *Miniopterus schreibersii* (n = 95), *Rhinolophus ferrumequinum* (n = 16), *R*. *hipposideros* (n = 12). Bat species with small sample size (n<5) were excluded from the latter analysis. The COIN (Conditional Inference Procedures in a Permutation Test Framework) package was used to correct P values of linked parameters. Differences were considered significant when P<0.05.

**Table 1:** Tick species and stages collected in Hungary, shown according to their bat hosts. Five females and two nymphs of *I*. *ariadnae*, which were collected from cave walls (Ariadne Cave System), are not included.

Tick		Bat (number of ticks per number of bats)											
		Vespertilionidae									Rhinolophidae		Miniopteridae
Species	Stage	MALC	MBEC	MNAT	MEMA	MDAU	MDAS	MMYO	PAUR	BBAR	RHIP	REUR	MSCH
***Ixodes ariadnae***	larva	4/2	3/3	1/1	6/6	5/3	5/2	-	1/1	-	1/1	-	-
	nymph	-	4/4	-	4/3	-	-	1/1	3/3	-	-	-	-
	female	-	-	-	-	-	-	-	-	-	-	-	-
***Ixodes vespertilionis***	larva		1/1	-	-	-	-	-	1/1	-	8/7	-	-
	nymph	-	-	-	-	-	-	-	-	-	6/4	-	-
	female	-	-	-	-	-	-	-	-	-	2/1	1/1	-
***Ixodes simplex***	larva	-	-	-	-	-	-	-	-	1/1	-	-	23/10
	nymph	-	-	-	-	-	-	-	-	-	-	-	11/10
	female	-	-	-	-	-	-	-	-	-	-	-	4/4

Abbreviations: MALC- *Myotis alcathoe*, MBEC—*My*. *bechsteinii*, MNAT—*My*. *nattereri*, MEMA—*My*. *emarginatus*, MDAU—*My*. *daubentonii*, MDAS—*My*. *dasycneme*, MMYO—*My*. *myotis*, PAUR—*Plecotus auritus*, BBAR—*Barbastella barbastellus*, RHIP—*Rhinolophus hipposideros*, REUR—*R*. *euryale*, MSCH—*Miniopterus schreibersii*.

**Table 2:** Tick species and stages collected in Romania, shown according to their bat hosts.

Tick		Bat (number of ticks per number of bats)								
		Vespertilionidae					Rhinolophidae			Miniopteridae
Species	Stage	MNAT	MCAP	MDAU	MBLY	ESER	REUR	RFER	RMEH	MSCH
***Ixodes vespertilionis***	larva	1/1	9/1	28/16	4/1	2/2	-	26/9	7/4	2/2
	nymph	-	4/2	9/8	2/2	-	-	6/6	-	-
	female	-	1/1	1/1	-	1/1	1/1	1/1	-	-
***Ixodes simplex***	larva	-		-	-	-	-	-	-	56/33
	nymph	-		-	-	-	-	-	-	39/33
	female	-		-	-	-	-	-	-	5/5

Abbreviations: MNAT—*Myotis nattereri*, MCAP—*My*. *capaccinii*, MDAU—*My*. *daubentonii*, MBLY—*My*. *blythii*, ESER—*Eptesicus serotinus*, REUR—*Rhinolophus euryale*, RFER—*R*. *ferrumequinum*, RMEH—*R*. *mehelyi*, MSCH—*Miniopterus schreibersii*.

### Ethical approval

Authorization for bat capture was provided by the National Inspectorate for Environment and Nature in Hungary (No. 14/2138-7/2011). Bat banding license numbers are TMF-14/32/2010 (DK), 59/2003 (PE) and TMF-493/3/2005 (TG), 65/2003 (SAB). Bats were handled according to the current law of animal welfare regulation (1998. XXVIII.). Permission from the Institutional Animal Care and Use Committee (IACUC) was not necessary, because bats were released in the field after tick removal (none taken to participating Institutes).

## Results

### Tick infestations of bats

In the two countries 308 ixodid ticks have been collected from 200 individuals of 17 bat species (Table 1, Table 2). *Ixodes ariadnae* was represented by 45, *I*. *vespertilionis* by 124 and *I*. *simplex* by 139 specimens (larvae, nymphs and females). In Hungary *I*. *ariadnae* was significantly more frequently found on bat species in the family Vespertilionidae, whereas *I*. *vespertilionis* was associated with Rhinolophidae (P<0.00001). *Ixodes ariadnae* was not collected in Romania, where *I*. *vespertilionis* occurred usually on representatives of both Vespertilionidae and Rhinolophidae (Table 2). Discounting one larva collected from *Barbastella barbastellus*, *I*. *simplex* was exclusively found on *Mi*. *schreibersii*.

In general, there was no significant difference between the intensity of tick infestation between bat species (p = 0.4279, df = 5, χ^2^ = 4.9026), but there was a significant difference in the intensity of infestation of bats with different tick stages (larva: n = 155; nymph: n = 79; female: n = 13; p = 0.0005, df = 2, χ^2^ = 14.924), i.e. ixodid tick larvae occurred in highest individual number on their hosts. In the case of *I*. *ariadnae* or *I*. *vespertilionis* there was no significant difference in the intensity of infestation between bat species, i.e. between *My*. *bechsteinii* and *My*. *emarginatus* (p = 0.4497, W = 28) or between *My*. *daubentonii*, *R*. *ferrumequinum* and *R*. *hipposideros* (p = 0.8719, df = 2, χ^2^ = 0.27423), respectively (Table 1, Table 2). Similarly, concerning these five bat species, there was no significant difference between intensities of their infestations with different tick stages, except for *My*. *daubentonii* and *R*. *ferrumequinum* on which larvae occurred significantly more frequently than nymphs/females (p = 0.02013, df = 2, χ^2^ = 7.8106). Similarly, infestation of *Mi*. *schreibersii* with *I*. *simplex* had the highest intensity when larvae were present on bats (larva: n = 79; nymph: n = 50; female: n = 9; p = 0.001674, df = 2, χ^2^ = 12.786).

### DNA of piroplasms in bat ticks

DNA sequences of piroplasms were detected in 20 bat ticks (Table 3). *Ixodes simplex* carried piroplasm DNA significantly more frequently (13 of 138 specimens), than *I*. *vespertilionis* (3 of 124 specimens) (P = 0.02). The largest variety of *Babesia* and *Theileria* DNA sequences was also shown to be present in *I*. *simplex* (Table 3).

**Table 3:** Results of molecular analyses of bat ticks for the presence of piroplasms.

*Ixodes*species	Tick stage or sex	PCR positive / all analysed ticks	Results of sequencing(length, % identity, sample number)	Bat host of PCR positive ticks^#^	Location(s) of PCR positive ticks in Fig 1	Reference sequence	Accession number of sequence in this study (name of isolate)
*I*. *ariadnae*	larva	4/26	*Babesia vesperuginis* (448 bp, 100%, 4×)	MDAS	1	AJ871610	KU958544 (Ia-Bv-1)
	nymph	0/14	-	-	-	-	-
	female	0/5	-	-	-	-	-
*I*. *vespertilionis*	larva	3/89	*Babesia vesperuginis* (448 bp, 100%, 2×)	ESER, MDAU	2	AJ871610	KU958544 (Ia-Bv-1)
			*Babesia crassa* (410 bp, 98.5%, 1×)	RHIP	3	KF791205	KU958546 (Iv-Bcr-1)
	nymph	0/27	-		-	-	-
	female	0/8	-		-	-	-
*I*. *simplex*	larva	8/79	*Babesia crassa* (410 bp, 98.3%, 1×)	MSCH	4	KF791205	KU958545 (Is-Bcr-1)
			*Babesia venatorum*-like (105 bp, 100%, 1×)	MSCH	2	KC007118	KU958553 (Is-Bv-1)
			*Babesia canis* (420 bp, 100%, 1×)	MSCH	2	JF461253	KU958552 (Is-Bca-2)
			*Theileria capreoli* (425 bp, 99.5%, 1×)	MSCH	4	KJ188219	KU958547 (Is-Tc-1)
			*Theileria orientalis* (432 bp, 100%, 4×)	MSCH	2, 4, 5	AB668373	KU958549 (Is-To-1)
	nymph	4/50	*Babesia crassa* (410 bp, 98.5%, 1×)	MSCH	2	KF791205	KU958546 (Iv-Bcr-1)
			*Babesia canis* (420 bp, 100%, 1×)	MSCH	2	KC902833	KU958551 (Is-Bca-1)
			*Babesia canis* (420 bp, 100%, 2×)	MSCH	2	JF461253	KU958552 (Is-Bca-2)
	female	1/9	*Theileria* sp. OT3 (432 bp, 100%, 1×)	MSCH	4	DQ866839	KU958550 (Is-TOT3-1)

^**#**^Abbreviations: MDAS—*Myotis dasycneme*, ESER—*Eptesicus serotinus*, MDAU—*M*. *daubentonii*, RHIP—*Rhinolophus hipposideros*, MSCH—*Miniopterus schreibersi*

In *I*. *ariadnae* only a DNA sequence of *B*. *vesperuginis* (identity: 448/448 bp = 100%) was shown to be present. All four PCR-positive larvae were removed from the same bat. In *I*. *vespertilionis* larvae sequences of *B*. *vesperuginis* (identity: 448/448 bp = 100%) and *B*. *crassa* (identity: 404/410 bp = 98.5%) were detected (Table 3).

Adding to the presence of the latter sequence in *I*. *simplex* nymphs, in a larva of this tick species the sequence of another genotype of *B*. *crassa* (identity: 403/410 = 98.3%) was demonstrated (Table 3), which was not detected before in Hungary. From *I*. *simplex* a shorter sequence of the zoonotic *B*. *venatorum* (identity: 105/105 bp = 100%) was also amplified, showing less identity with other piroplasms (second closest to *B*. *occultans* and *T*. *equi*, with 103/105 bp = 98.1% identity). In *I*. *simplex* larvae/nymphs two sequences of *B*. *canis* (both identities: 420/420 bp = 100%) were also detected.

Results of sequencing demonstrated DNA of two *Theileria* spp. exclusively in *I*. *simplex* larvae. These were *T*. *capreoli* (identity: 423/425 bp = 99.5%) and *T*. *orientalis* (identity: 432/432 bp = 100%). In addition, one female *I*. *simplex* carried the sequence of *Theileria* sp. OT3 (identity: 432/432 bp = 100%) (Table 3).

**Fig 2:**
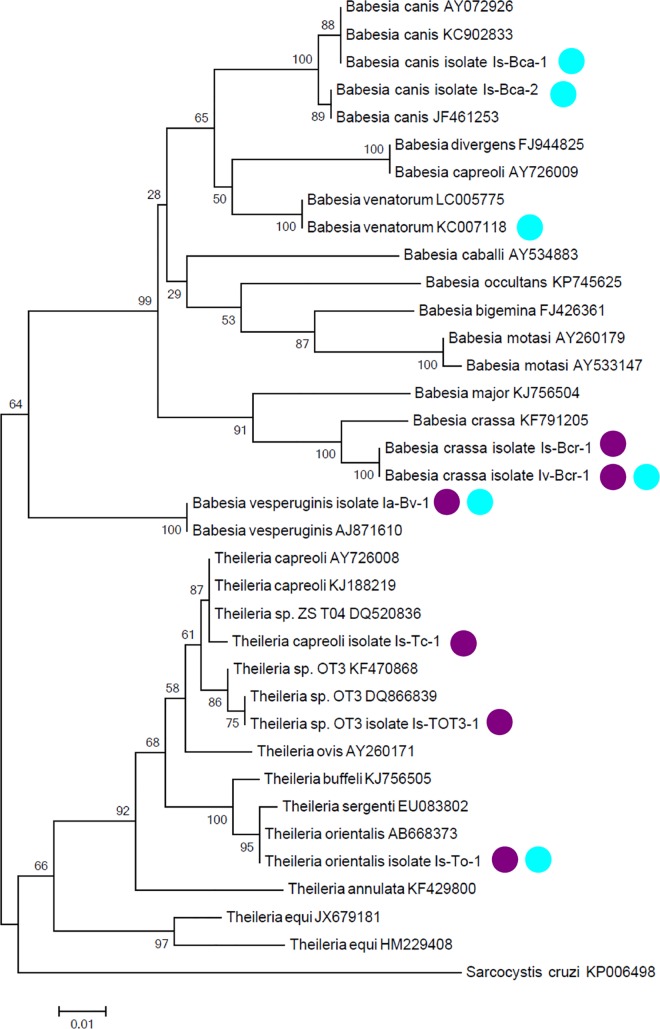
Phylogenetic relationships of 18S rDNA sequences of piroplasms identified in this study and relevant sequences previously deposited in GenBank. Sequences identified in the present study in Hungary and in Romania are highlighted with purple or turquoise dots, respectively. The shorter sequence of *Babesia venatorum* from this study is not included, therefore its reference sequence (to which it showed 100% identity) is marked. Branch lengths correlate to the number of substitutions inferred according to the scale shown.

In the phylogenetic analysis, all sequences of *Babesia* and *Theileria* spp. amplified from bat ticks in the present study clustered together with relevant genotypes available in GenBank (and published from previously known "type" hosts of these piroplasms) (Fig 2). Their separation from other piroplasms was confirmed by high bootstrap values (Fig 2). Taken together, piroplasm sequences were demonstrated in bat ticks from three places of Hungary and two places of Romania (Table 3, Fig 1); three sequences of piroplasms were detected only in samples from Hungary, three of them only in Romania and three in both countries (Fig 2).

## Discussion

In this study all three ixodid tick species have been collected, which are specialized to bat hosts in Europe. Among them, *I*. *ariadnae* is known to occur in three countries of Central and Western Europe (Hungary, Germany and Belgium: [19]), whereas *I*. *vespertilionis* and *I*. *simplex* are more widespread on the continent (the latter predominantly south of latitude 49° N: [20]). In the present study *I*. *ariadnae* was not found in Romania, suggesting that the bat tick fauna in this country is similar to that in the Balkans, with the predominance of *I*. *simplex* [21]. A plausible explanation for this phenomenon is the absence of regular bat migration between the mountainous regions of Hungary and Romania, which otherwise could have caused the spread of *I*. *ariadnae* towards the southeast (the main route of long distance bat migration in the region is in the southwestern-northeastern direction: [22]).

The present results confirmed that the preferred hosts of *I*. *ariadnae* belong to Vespertilionidae [11, 19], those of *I*. *vespertilionis* to Rhinolophidae, while *I*. *simplex* is adapted to parasitize *Mi*. *schreibersii* (Miniopteridae) [23]. Nevertheless, to the best of our knowledge, several new host associations of ixodid bat ticks are reported here for the first time. In particular, *I*. *ariadnae* was found newly on four *Myotis* spp., *I*. *vespertilionis* on two *Myotis* spp., as well as on *Eptesicus serotinus* and *Rhinolophus mehelyi*, finally *I*. *simplex* on *Barbastella barbastellus* (Table 4).

**Table 4:** Host associations of ixodid bat ticks reported previously and in this study.

	Host species reported previously	References	New host species in this study
***Ixodes ariadnae***	*Myotis alcathoe*	[11, 19]	*Myotis dasycneme*
	*Myotis bechsteinii*		*Myotis daubentonii*
	*Myotis blythii*		*Myotis emarginatus*
	*Myotis myotis*		*Myotis nattererii*
	*Plecotus auritus*		
***Ixodes vespertilionis***	*Miniopterus schreibersii*	[9, 21, 23, 24, 25, 26, 27, 28]	*Eptesicus serotinus*
	*Myotis bechsteinii*		*Myotis capaccinii*
	*Myotis blythii*		*Myotis dasycneme*
	*Myotis brandtii*		*Rhinolophus mehelyi*
	*Myotis daubentonii*		
	*Myotis emarginatus*		
	*Myotis myotis*		
	*Myotis mystacinus*		
	*Myotis nattererii*		
	*Plecotus auritus*		
	*Rhinolophus euryale*		
	*Rhinolophus ferrumequinum*		
	*Rhinolophus hipposideros*		
***Ixodes simplex***	*Miniopterus schreibersii*	[21, 23, 25, 29]	*Barbastella barbastellus*
	*Myotis alcathoe*		
	*Rhinolophus euryale*		
	*Rhinolophus ferrumequinum*		

The intensity of tick infestation was not significantly different between small and large size bat species (in the case of *My*. *daubentonii* vs. *R*. *ferrumequinum*, respectively), suggesting that factors depending on body size (such as the body surface area, interrelated with metabolic rate, heat emission: [30]) may not be crucial for host finding by bat ticks. Similarly, it has been reported that body size of passeriform bird species did not significantly influence the intensity of their tick infestation [31]. On the other hand, the intensity of infestation with bat tick larvae was significantly higher, than with later stages in the life cycle. This finding is consistent with the significant decrease of individual number of tick stages with the advance of tick developmental cycle [32].

Taking into account the considerable lack of data in literature on the vector potential of ixodid bat ticks, and the recent finding of *B*. *canis* DNA in bat faeces [5], DNA extracts of 307 specimens were molecularly analysed for the presence of piroplasms (Apicomplexa: Piroplasmida). Among piroplasms, *Babesia* species are known to be transmitted transovarially by female ticks to the next generation (i.e. to larvae prior to their blood meal), whereas *Theileria* species are transmitted transstadially [33]. The latter implies that there is no other way for tick larvae to harbor theileriae or to contain theileria DNA, than to ingest these with the blood meal from a host/reservoir which is either theileria-infected or at least theileria DNA is present in its blood stream.

*Babesia vesperuginis* DNA was molecularly identified here in *I*. *ariadnae* and *I*. *vespertilionis*. This piroplasm is pathogenic to bats, and was reported to infect *Pipistrellus pipistrellus* [34], several *Myotis* spp. (including *My*. *daubentonii*, on which bat species a PCR positive tick was collected in the present study) as well as *Plecotus auritus* [35]. Taking into account that soft ticks (*Argas vespertilionis*) have been incriminated as vectors of *B*. *vesperuginis* [34], the present results suggest that bat ticks carrying this piroplasm (or its DNA) ingested it with the blood meal, i.e. further *Myotis* spp. (exemplified by *My*. *dasycneme*) and *Eptesicus serotinus* might also be susceptible to *B*. *vesperuginis*.

*Babesia crassa* has low pathogenicity in small ruminants and its vector is unknown [36]. This piroplasm (or closely related genotypes) were reported to occur only in the Middle-East, but recently one genotype has also been identified in *Haemaphysalis inermis* ticks in Central Europe, Hungary [37]. In the present study two different DNA sequences of *B*. *crassa* were detected in bat ticks (*I*. *vespertilionis*, *I*. *simplex*) in both Hungary and Romania. These two bat tick species have never been reported from small ruminants, and therefore their PCR positivity can be explained by ingesting *B*. *crassa* DNA-containing blood meal from bats. In this context it may be epidemiologically relevant that *B*. *crassa* was reported to be present in *H*. *sulcata* [38], and this tick species was reported to infest bats in the larval stage and small ruminants in the adult stage [39].

*Babesia canis* is an important parasite of dogs. Wild canids are also susceptible [40]. The known vector of this piroplasm is *Dermacentor reticulatus*, which is a tick species seldom infesting bats, including *Mi*. *schreibersii* [8]. Recently, bats were reported to pass the DNA of *B*. *canis* in their faeces [5]. Taking into account that it is very unlikely that relevant lineages of *I*. *simplex* (found to be PCR positive here) had become infected from canids (from which hosts *I*. *simplex* has never been reported) in a previous stage or generation, *B*. *canis* or its DNA might have been present in the blood of relevant bats. This possibility is supported by recent finding of *B*. *canis* DNA in bat tissues [41].

Interestingly, the DNA of *B*. *venatorum* was amplified from one larva of *I*. *simplex* in Romania. Although the sequence was 100% identical with *B*. *venatorum* and differed from other piroplasms, because of its shortness no final conclusion can be drawn on the occurrence of *B*. *venatorum* DNA in bat ticks. This piroplasm (associated with cervids as hosts) is zoonotic, with *I*. *ricinus* as its vector. It is noteworthy that *I*. *ricinus* occurs on bats (e.g. [9]), and the present results suggest that this may allow bats to become carriers of *B*. *venatorum* or its DNA (taking into account that *I*. *simplex* has never been reported from cervids or from humans). On the other hand, the host of *I*. *simplex*, *Mi*. *schreibersii* may live in large colonies in the human environment (e.g. mines, man made tunnels, ruins: [20]). Therefore, this preliminary finding deserves further molecular epidemiological investigation.

Among *Theileria* spp. and genotypes, the DNA of *Theileria* sp. OT3 has been detected here in a female *I*. *simplex*. This piroplasms (with unknown pathogenicity) was formerly reported to infect small ruminants in Italy [42], but recently its DNA has also been reported from *Haemaphysalis punctata* in northern Hungary [37], and this tick species is known to infest bats [10].

In addition, the DNA of two *Theileria* spp. have been shown here to be present in larvae of *I*. *simplex* from *Mi*. *schreibersii*. Among them, *T*. *capreoli* is a mildly pathogenic parasite of cervids. The tick species *H*. *concinna*, in which the DNA of *T*. *capreoli* has been recently demonstrated in Hungary[37], is also known to occasionally infest bats [43].

Members of the *T*. *orientalis* complex (*T*. *orientalis*, *T*. *buffeli*) infect cattle in the tropical-subtropical regions of the globe, usually with low pathogenicity. Recently, *T*. *orientalis* has been shown to emerge in Central Europe [37] and Australia [44], sometimes severely affecting cattle [45]. Vectors of the *T*. *orientalis* complex are *Haemaphysalis* spp. [33]. *Haemaphysalis* spp. may accidentally infest bats, and in particular *H*. *punctata*, the most likely vector of *T*. *orientalis* in Europe, was synonymously called "*H*. *rhinolophi*" [10]. Furthermore, in South-East Asia (where species of the *T*. *orientalis* complex are widespread) at least one *Haemaphysalis* sp. has bats as preferred hosts [46]. These literature data attest a possible connection between large ruminants and bats via *Haemaphysalis* sp. ticks. In the present study only bat tick (*I*. *simplex*) larvae were PCR positive for *T*. *capreoli* and *T*. *orientalis*. This means that relevant piroplasms (or their DNA) could have been acquired by the larvae exclusively from the blood of bat hosts, because there is no transovarial, only transstadial transmission in the case of theileriae [33]. The significance of the potential epidemiological role of bats in bovine theileriosis deserves further attention, as several bat species may use cattle stables for roosting [47].

In summary, competent vectors of the above piroplasms (that have been hitherto reported from hosts other than bats) are *D*. *reticulatus*, *I*. *ricinus* and *Haemaphysalis* spp. These tick species are rarely found on bats, most likely attaching to bats when roosting in nests of small mammals (e.g. in tree holes) [5], or when gleaning bat species feed on insects from the lower vegetation in meadows or forests. However, *Mi*. *schreibersii*, associated with most of the piroplasms identified in the present study, is not known to forage on the ground level [48]. Alternatively, blood-sucking flies have the potential to carry and transmit *Babesia* spp. [49] and *Theileria* spp. [50], and flies (Insecta: Diptera) are among the frequent food items of e.g. *Mi*. *schreibersii* [51]. This implies that bats may get into contact with or may have access to piroplasms or piroplasm DNA from their food.

Thus, there are two plausible explanations for the above, unexpected findings. The first is that bats get into frequent contact with the DNA of vector-borne pathogens contained in their food. During digestion this DNA may pass through the gut wall (barrier) un- or only partly digested, thus appearing in the circulation (or perhaps other tissues) from where bat ticks can take it up with their blood meal. In support of this possibility, it has recently been verified that meal-derived DNA fragments (even long ones) can avoid degradation and through not-yet-known mechanisms enter the circulation, at least in humans [52].

Another, although less likely explanation is that bats are susceptible to a broader range of piroplasms than previously thought. The phylogeny of piroplasms has recently been shown to reflect considerable host diversity and limited host specificity [53], suggesting that these tick-borne protozoa have undergone frequent host switches during their evolution. In this context the present results may imply that bats may share piroplasms with a broad range of mammals (from various orders). Similarly, several *Babesia* and *Theileria* spp. are known to infect hosts from different mammalian orders (e.g. *B*. *caballi*, *B*. *canis*, *B*. *divergens*, *B*. *microti*, *T*. *equi*: [53]).

## Conclusions

Bat ticks are not known to infest dogs or ruminants, i.e. typical hosts and reservoirs of piroplasms molecularly identified in *I*. *vespertilionis* and *I*. *simplex*. Therefore, DNA sequences of piroplasms detected in these bat ticks most likely originated from the blood of their respective bat hosts. This may indicate that either bats are susceptible to a broader range of piroplasms than previously thought, or at least the DNA of piroplasms may pass through the gut barrier of bats during digestion of relevant insect vectors. In light of these findings, the role of bats in the epidemiology of piroplasmoses deserves further investigation.

## Supporting Information

S1 Table Geographical coordinates of collection sites in the present study. Letters: A—Ariadne Cave System and caves in the Pilis Mountains, B—Bükk Highlands Cave System (see Fig 1).
(DOC)
