# Supplementary material for: DNA of Piroplasms of Ruminants and Dogs in Ixodid Bat Ticks
Source: PLoS One. 2016 Dec 8;11(12):e0167735. doi: 10.1371/journal.pone.0167735 (PMC5145180; doi:10.1371/journal.pone.0167735)
Supplement: S1 Table — Letters: A—Ariadne Cave System and caves in the Pilis Mountains, B—Bükk Highlands Cave System (see Fig 1). (DOC) [file pone.0167735.s001.doc]

**S1 Table. Collection sites of the present study.** Letters: A - Ariadne Cave System and caves in the Pilis Mountains, B - Bükk Highlands Cave System (see Figure 1).

| **Country** | **Location** | **Northern latitude** | **Eastern longitude** |
| --- | --- | --- | --- |
| Hungary | Ajándék Cave (A) | 47.7005 | 18.8467 |
|  | Baradla Cave | 48.4667 | 20.5 |
|  | Béke Cave | 48.4607 | 20.5428 |
|  | Büdöskúti Cave | 46.1242 | 18.1829 |
|  | Esztergom | 47.7856 | 18.7403 |
|  | Fekete Cave (B) | 48.1054 | 20.6593 |
|  | Felsőtárkány | 47.973 | 20.417 |
|  | Jáspis Cave (B) | 48.1075 | 20.5812 |
|  | Jávorkúti Cave (B) | 48.1021 | 20.5770 |
|  | Láner Cave (B) | 48.1007 | 20.5802 |
|  | Legény Cave (A) | 47.6994 | 18.8442 |
|  | Létrási Cave (B) | 48.1011 | 20.5803 |
|  | Lökvölgyi Cave (B) | 48.0618 | 20.4604 |
|  | Mád | 48.1898 | 21.2765 |
|  | Mánfai Cave | 46.1584 | 18.2404 |
|  | Mátraszentimre | 47.9098 | 19.8757 |
|  | Miskolc | 48.1042 | 20.7914 |
|  | Parád | 47.922 | 20.044 |
|  | Pénzpatak Cave (B) | 48.0535 | 20.5472 |
|  | Szársomlyó | 45.8564 | 18.4111 |
|  | Szepesi Cave (B) | 48.1007 | 20.5803 |
|  | Szivárvány Cave (B) | 48.1018 | 20.5519 |
|  | Szoplaki Cave (A) | 47.7081 | 18.875 |
|  | Viszló | 48.4928 | 20.8878 |
| Romania | Tulcea | 45.1833 | 28.6667 |
|  | Gura Dobrogei | 44.4811 | 28.5275 |
|  | Avenul Betfia | 46.9744 | 22.0157 |
|  | Avenul de la Păuleasa | 45.0163 | 21.8713 |
|  | Pecinișca | 44.8567 | 22.4143 |
|  | Roșia | 46.3061 | 23.1303 |
|  | Canaraua Fetii | 44.0533 | 27.6742 |
